# Thyroid Hormone Metabolites Quantified in Pup and Adult Rat Cerebellum, Cortex and Whole-Brain Samples Using an Automated Online SPE-LC-MS/MS Method

**DOI:** 10.3390/metabo14010061

**Published:** 2024-01-17

**Authors:** Christiane Hindrichs, Tilmann Walk, Robert Landsiedel, Hennicke Kamp, Steffen Schneider, Stephanie Melching-Kollmuss, Dorothee Funk-Weyer

**Affiliations:** 1 BASF Metabolome Solutions GmbH, Tegeler Weg 33, 10589 Berlin, Germany; christiane.hindrichs@basf.com (C.H.); tilmann.walk@basf.com (T.W.); 2 Department of Chemistry, Rheinland-Pfälzischen Technischen Universität Kaiserslautern-Landau, Erwin-Schrödinger-Straße 52, 67663 Kaiserslautern, Germany; 3 Experimental Toxicology and Ecology, BASF SE, Im Spitzenbusch 10, 67227 Frankenthal, Germanysteffen.schneider@basf.com (S.S.);; 4 Pharmacology and Toxicology, Institute of Pharmacy, Free University of Berlin, 14195 Berlin, Germany; 5 Agricultural Solutions, BASF SE, Speyerer Str. 2, 67117 Limburgerhof, Germany

**Keywords:** thyroid hormones, brain, cerebellum, cortex, rat, automated SPE, LC-MS/MS

## Abstract

Changes in thyroid hormone (TH) levels in rat brain at early developmental stages are correlated with adverse effects on offspring development. To characterize the ability of substances to interfere with the TH concentrations in, e.g., rat brain, it is essential to know the mean TH concentrations in this tissue under control conditions. In this publication, an online solid-phase extraction (SPE) liquid chromatography (LC) tandem mass spectrometry (MS/MS) method was validated and used to measure TH metabolites (T4, T3, rT3, T2 and T1) in the brains of untreated rats. Data on TH concentrations in the whole brain and separate data from the cerebellum and the cortex are shown. The corresponding samples were gathered from young rats at postnatal days (PND) 4 and 21/22 and from adult rats. The results show inter alia the high accuracy and precision of the method, and LOQs of 0.02 ng/mL were determined for T1, T2 and rT3 and of 0.15 ng/mL for T3 and T4. Technical variability is low, as shown by the relative standard deviations of 7.5–20%. For our rat model, we found that T4, T3 and T2 concentrations rise from PND4 to PND21, whereas the rT3 concentration decreases; as well as there is no statistical difference between TH concentrations in the male and female rat brain. This method is suitable to analyze TH metabolites in the brain and build up a database of historical TH concentrations in control rats. Together, this yields a robust diagnostic tool to detect potentially adverse disturbances of TH homeostasis in the most vulnerable anatomic structure.

## 1. Introduction

TH levels in mammals are regulated by a complex system, the hypothalamic–pituitary–thyroid axis (HPT axis). Neurons in the paraventricular nucleus (PVN) of the hypothalamus synthesize and release thyrotropin-releasing hormone (TRH) into the anterior pituitary, which releases the thyroid-stimulating hormone (TSH). TSH stimulates the thyroid gland to produce thyroxine (T4) and the biologically active triiodothyronine (T3) and to release them into the circulation. THs enter different tissues via transporters, for example, monocarboxylate transporter 8 (MCT8) or organic anion transporter polypeptide 1C1 (OATP1C1), and they cross the blood–brain barrier (BBB) of the choroid plexus [1]. According to [2], T4 is predominantly transported compared to T3 into astrocytes in the central nervous system (CNS) due to the higher affinity of the transporter towards T4 [2]. In the astrocytes, around 80% of T3 is locally formed from T4 by deiodinase 2 (DIO2) [3], whereas DIO3 inactivates both T4 to rT3 and T3 to T2 [1]. T3 transfers into neuronal cells via MCT8 and binds to its nuclear receptor (thyroid hormone receptor, TR), initiating target gene transcription [1]. Disturbed TH homeostasis can affect the developing brain from the fetal stage to young adulthood. This has been correlated to a decrease in the child’s gray matter volume and the child’s IQ [4]. Also, in animal studies, thyroid hormone concentrations are usually determined in adult or offspring blood. From there, thyroid hormone changes in the offspring brain are assumed without having the respective data. Therefore, having a robust analytical method to detect TH concentrations in the brain of the fetus will be a critical parameter for the evaluation of brain development. This is reflected in existing adverse outcome pathways (AOPs), where the causal key event of “TH changes in target tissue” follows the key event of “serum TH changes” [5,6]. Total TH concentrations in (maternal) blood are relatively straightforward to measure but may not exactly reflect the actual concentrations in developing brains of fetuses and young animals. The analysis of THs in the brain is, however, demanding.

The published TH analyses in developing rat brain address general method developments and improvements [7,8,9], deficiency studies [10,11] and substance administration [12,13]. Ford et al. [9] optimized a method for rat brain TH measurement to minimize confounding effects through the phospholipid matrix and reach a low detection limit. On postnatal day 2 (PND2), the forebrains from Long-Evans rat pups were isolated, and on PND6 and -14, their whole brains were removed. The experimental method quantification limits (MQLs) were set at 0.105, 0.040 and 0.090 ng/g for T3, rT3 and T4 [9]. In another publication, a forebrain TH analysis was conducted in rat pups on PND0, -2 and -6 by liquid chromatography tandem mass spectrometry (LC-MS/MS), yielding a limit of quantification (LOQ) of 0.1 ng/g brain for T3 and T4 and in serum of 0.1 ng/mL [14].

With regard to commonly used sampling time points in animal studies, we developed an online solid-phase extraction liquid chromatography tandem MS (abbreviated as xLC-MS/MS) for the quantitative analysis of TH concentrations in rat brain. Here, we describe TH analysis in the whole brain, cerebellum and cortex of untreated rats on PND4, -21/22, -71–72 and -97–99 (the latter are referred to as adult rats). TH concentrations in the brains of rats at different ages, taken from control groups of different studies, help to define “normal” TH concentration ranges and set up an in-house database. This will allow the identification of treatment-related changes in TH concentrations in the brain and in different brain compartments. The knowledge of biological variation of physiological levels at relevant time points will allow us in the future to better assess thyroid hormone-related neurodevelopmental toxicity at a later and more decisive step in an AOP. 

## 2. Materials and Methods

### 2.1. Materials

#### 2.1.1. Analytes

The thyroid hormones 3,3′,5,5′-tetraiodo-L-thyroxine (T4), 3,3′,5-triiodo-L-thyronine (T3), 3,3′,5′-triiodo-L-thyronine (reverse T3; rT3) and 3,3′-diiodo-L-thyronine (T2) and its isotope-labeled standard ^13^C_6_-T2 were purchased from Sigma-Aldrich (St. Louis, MO, USA). From Toronto Research Chemicals (Toronto, ON, Canada, M3J 2J8), 3-monoiodo-L-thyronine (T1) as well as its isotope-labeled standard (^13^C_6_-T1) were obtained. The isotope-labeled standards ^13^C_6_-T4, -rT3 and -T3 were purchased as a concentrated solution of 100 µg/mL in 0.1 N NH_3_ in methanol (MeOH) (*v*/*v*) from Cerilliant, a Sigma Aldrich Company (Round Rock, TX, USA, 78665). All used solvents were of “LC-MS CHROMASOLV™ ≥ 99.9%” grade (Honeywell Specialty Chemicals Seelze GmbH, Seelze, Germany), and water was taken from a Milli-Q^®^ station (Merck KGaA, Darmstadt, Germany). 

#### 2.1.2. Standard Solutions

Stock solutions of the thyroid hormones were firstly prepared in dimethyl sulfoxide (DMSO), and further serial standard dilutions were generated in 70% aqueous MeOH. The final standard-concentration samples for measurement were prepared from standard dilutions in 70% aqueous MeOH, and 20 µL internal standard was added. The final volume was 140 µL. 

#### 2.1.3. Brain Samples

The untreated brain samples were taken from 4- and 21-day-old Wistar rats from different in-house studies conducted in 2015, 2022 and 2023 (samples received from a total of six different studies). The housing conditions included 12 h light and dark shifts at 20 to 24 °C, 45–65% humidity and 15 air changes per hour. The animals received food and drinking water ad libitum, both of which were regularly examined for possible contaminants. The pups themselves underwent daily examination for clinical symptoms. From studies one to four, 10 samples per sex and age (PND4, PND21 and 97–99 d) were taken, with the exception that from study two, the numbers of pups on PND4 were one male and six females. The amount of adult rat brains (71–72 d) from study five was divided between brain-region-specific and whole-brain analyses, for which 5 animals per sex and analysis were measured. Lastly, study six used pup samples for TH analysis of the cerebellum and the cortex, which resulted in 5 samples per sex, age (PND4 and -22) and brain region. 

### 2.2. Methods

#### 2.2.1. Sample Preparation

Prior to TH analysis, the rat brains were lyophilized for 3 days and afterwards homogenized by pulverization (Bead Ruptor Elite, biolab products, Bebensee, Germany). From the pulverized rat brain samples, a defined amount was weighed in depending on the age of the given rat samples. The following description of sample preparation is based on a previous method developed to measure THs in rat plasma, and this method was modified to analyze TH in rat brains [15]. From rat brains on PND21, 20 mg was taken; PND4 7 mg; and adult rats 40 mg; these samples were weighed in in a polypropylene Eppendorf tube (Safe-Lock Tubes, 2.0 mL, Eppendorf AG, Hamburg, Germany), 300 µL PBS was added and samples were sonicated for 5 min. Afterwards, 20 µL of the internal standard mix was added and mixed for 10 min at 14,000 rpm. The added internal standard mix contained ^13^C_6_-labeled internal standards (T4, T3, rT3, T2 and T1) to adjust to fluctuations during sample preparation and injection. For the following protein precipitation, 900 µL 1% formic acid (FA) in acetonitrile (ACN) was added, the mixtures were vortexed subsequently and then incubated for 30 min at −23 °C (±3 °C), whereas after 15 min the samples were vortexed a second time. Finally, the precipitates were transferred onto a 0.22 µm Ultrafree-CL filter (UFC40GV, Merck Millipore; Carrigtwohill, Co Cork, Ireland), while the used Eppendorf tubes and pipette tips were kept, and the loaded filters were centrifuged for 15 min at 5100 rpm at 4 °C. To remove the remaining precipitate from the vials’ walls, the kept Eppendorf tubes as well as the pipette tip were rinsed twice with 200 µL 80% aqueous ACN. After each time, the rinse was loaded onto the corresponding sample filter and centrifuged first for 6 min and second for 10 min at 5100 rpm at 20 °C. The final eluent was transferred into a 1 mL LC clear glass vial (Labsolute Th. Geyer GmbH & CoKG, Renningen, Germany), evaporated to dryness at 40 °C, resuspended in 140 µL 70% aqueous MeOH, vortexed and sealed with a silicone Micromat (Thermo Fisher Scientific, Langerwehe, Germany), and a volume of 112.5 µL was injected for analysis. 

#### 2.2.2. Solid-Phase Extraction (SPE) and Liquid Chromatography (LC)

A Symbiosis Pharma System (Spark Holland, Emmen, The Netherlands) was used, and Oasis™ on-line SPE hydrophilic–lipophilic balance (HLB) cartridges (10 × 2 mm, 30 µm, Waters, Eschborn, Germany) were selected for SPE. Conditioning and equilibration of the cartridges were with 1000 µL MeOH and 1000 µL Millipore water, each at a flow rate of 5 mL/min. A sample load of the 112.5 µL injected sample was accompanied by 500 µL 0.5% FA in H_2_O, followed by washing of the cartridges with 1000 µL 1:1 MeOH:0.5% FA H_2_O. To elute the analytes from the sorbent of the cartridges, the high-pressure dispenser (HPD) focusing mode was used. Thereto, 200 µL of MeOH at 0.1 mL/min was used to elute the analytes, which were transported onto the LC column where they encountered the LC pump flow. At the beginning of the gradient, the LC flow was 100% aqueous to enrich the analytes at the beginning of the LC column, which was a Raptor biphenyl column (2.7 µm, 50 × 2.1 mm, Restek, Bellefonte, PA, USA). The LC pump flow consisted of two mobile phases (MP): mobile phase A (MP-A) consisted of 0.1% acetic acid (AA) in H_2_O and mobile phase B (MP-B) of 0.1% AA in MeOH. During elution mode, the LC flow was set at 0.1 mL/min the same as the HPD elution flow, and by termination of the elution, the LC flow rate increased to 0.3 mL/min and 5% MP-B. Over 1 min, the amount of MP-B increased further to 50%, then over 5 min to 70% and finally quickly to 100% MP-B in 30 s. This was held for around 2 min, after which re-equilibration was followed at 0% MP-B, and it took up to 15 min for one gradient to complete. An exemplary chromatogram can be seen in the supplementary materials in Figure S1.

#### 2.2.3. Instrumentation

Mass spectrometry (MS) analysis was performed on a Triple Quad 5500 Mass Spectrometer (MS, AB Sciex Instruments, Framingham, MA, USA) in positive ionization mode using multiple reaction monitoring (MRM). The optimized MS parameters are listed below (Table 1 and Table S1).

#### 2.2.4. Statistical Analysis

Statistical analysis and evaluation of the results were performed using GraphPad Prism software version 8.0.2 (GraphPad Software, San Diego, CA, USA). To determine statistical significance, the unpaired Welch *t*-test was performed, and the critical *p* value was set to 0.05.

## 3. Results

### 3.1. Method Validation

To guarantee sufficient specificity and robustness of the TH analysis of rat brains, this method was validated according to the DG Sante guidance document “SANTE/2020/12830”, from which the acceptance criteria in chapter 3 “Method validation parameters” were adopted [16]. Parameters included in the validation process were the coefficient of determination R^2^ (≥0.99); determination of THs at the corresponding limit of quantification (LOQ) with an accuracy of 100 ± 20%; and a relative standard deviation (RSD) ≤ 20%. Additionally, the possible carryover of certain analytes and their specificity were investigated. To verify the developed method regarding its robustness, specificity, and selectivity to quantify the analytes in the given matrix, a validation measurement was conducted. To quantify the analytes, analyzed standard curves were weighed 1/x and exceeded an R^2^ of ≥0.99, whereas the LOD covered the lowest standard concentration point. The accuracy and precision of the method were determined by analyzing five technical replicates of analytes’ LOQ concentration as well as their 10 x LOQ concentration. The yielded accuracies ranged from 90 to 110% with a precision range of 1.8–9.1%, which corresponded with the acceptance criteria [16] (see Table S2). The LOQ was ascertained to 0.02 ng/mL for T1, T2 and rT3 and to 0.15 ng/mL for T3 and T4. The carryover of the analytes was investigated by measuring two solvent blanks after the highest standard concentration. All results were lower than 30% of the analytes’ LOQ signal; therefore, a carryover effect could be excluded. Overall, it was concluded that the method quantified the analytes successfully in agreement with the underlying acceptance criteria.

### 3.2. Sample Preparation Is Robust for Brain Matrix

For normalization of the concentration of the analytes, control male and female rat samples were combined to generate a brain matrix pool. The pool contained 20 mg brain of each adult rat (*n* = 20). Technical replicates were prepared for each brain measurement to investigate consistency in sample preparation. All technical replicates were used to calculate the mean and standard deviation and to derive the RSD (%). The resulting RSDs (%) are listed in Table 2. Since they are lower than 20%, it is concluded that sample preparation is robust.

### 3.3. TH Concentrations in Rat Brain at Different Ages

**Figure 1:**
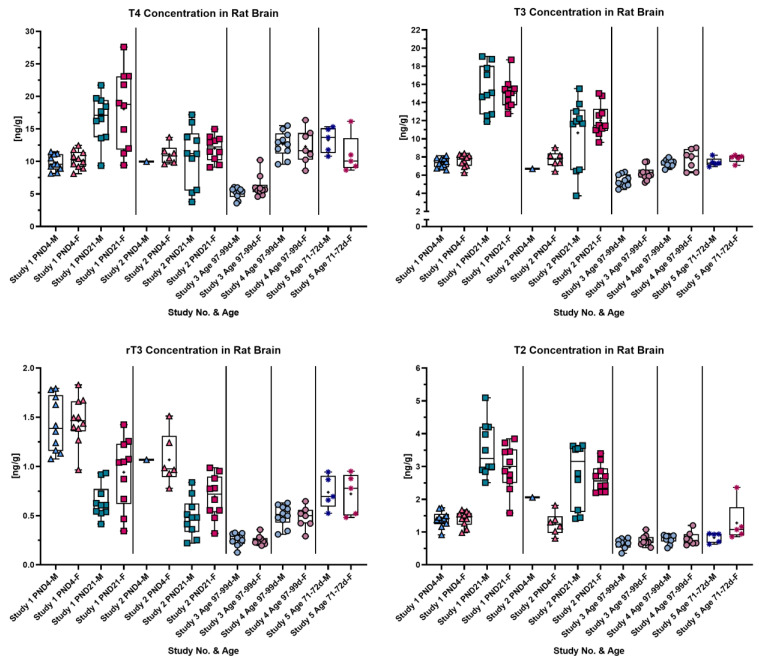
Each graph shows the concentration in rat brain at different ages of each analyte. Samples were derived from five studies, and age groups are differentiated by different symbols: triangles, PND4; squares, PND21; stars, 71–72 d; and dots, 97–99 d. The symbols are colored in blueish or reddish tones referring to male (blueish) or female (reddish) rats. In each box plot a “+” marks the mean of the data group. Notable is the increase in T4, T3 and T2 concentrations on PND21 compared to other age groups and the opposite concentration pattern over age of rT3.

Whole-brain analysis was conducted from rat brains at various ages, and the results are summarized in Figure 1. Overall, whole-brain samples were derived from five different studies, identified by distinguishing symbols on the graph: triangles denote PND4, squares PND21, stars 71–72-day-old rats and dots 97–99-day-old rats. From study two, only a limited number of samples from PND4 were received (males *n* = 1 and females *n* = 6); from study five, *n* = 5; and otherwise, 10 animals per sex were measured. Samples from studies three and four were taken in 2015 and stored in N_2_ at −80 °C. As seen in Figure 1, different TH concentrations were calculated from adult animals in studies three and four even though rats were at a similar age. Samples from the most recent study, study five, from adult rats (20 days younger than those from studies three and four) inserted themselves in the range of the results from study four. T2 concentration was unaffected between studies three to five, yielding results in a similar range throughout these studies. Notably, regarding T2, it had a low concentration close to the LOQ in adult rats and showed a markedly increased concentration on PND21. In general, on PND21, TH concentrations except rT3 sharply increased compared to the other age groups. TH concentrations on PND4 were similar to the ones found in adult rats. An opposite pattern to the one described above was detected for rT3 concentrations. rT3 concentrations in adult rats and on PND21 lay within a similar range, whereas on PND4, the highest concentrations were determined.

### 3.4. TH Concentrations in Rat Cerebellum and Cortex at Different Ages

**Figure 2:**
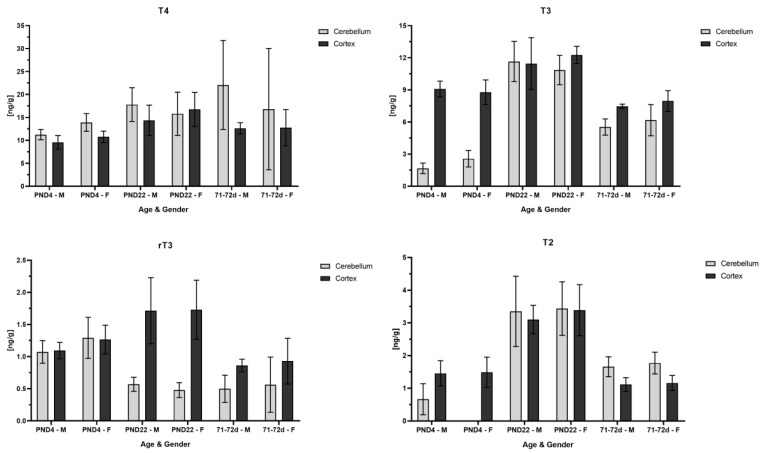
TH analysis in specific rat brain regions on PND4 and PND22 and in 71–72-day-old rats. The *Y* axis shows concentration in ng/g and the *x* axis male and female rats for each age group. The light-gray bar demonstrates the concentration found in cerebellum and the dark-gray bar in cortex. On PND22, T4, T3 and T2 concentrations yielded a similar concentration in both brain regions, whereas rT3 concentration sharply decreased in cerebellum as compared to cortex. On PND4, T3 concentration in cerebellum was one-third of the concentration found in cortex. T2 was not detected in cerebellum on PND4 in female rats and only in three out of six male rat pups.

In addition to the quantification of THs in the whole brain, an analysis of specific brain regions, the cerebellum and the cortex, was performed. Cerebellum and cortex samples were obtained from control rats on PND4 and PND22 and from 71–72-day-old rats. The results are displayed in Figure 2. Overall, it was observed that T4 concentrations were similar between the two regions within each age. Due to the higher standard deviation in the adult cerebellum, no definite statement is concluded regarding the similarity of T4 concentration on PND4 compared to PND22. On the contrary, T3 concentration was lower in the cerebellum than the cortex on PND4 but increased to a similar concentration as found in the cortex on PND22 and in adult rats. The analyte T2 followed the same pattern as the one mentioned for T3, whereby T2 was not detected at all in the female cerebellum samples and only in three of the six male cerebellums. A similar concentration in both regions on PND4 was found for rT3, whereas its concentration in the cerebellum decreased compared to the cortex on PND22 and in adult rats. Overall, both regional concentrations in the adult rats for T3, rT3 and T2 were lower and closer to the value found on PND4, with the exception that the rT3 concentration in the adult cerebellum was similar to the value found in the cerebellum on PND22. Further, T2 in the adult cerebellum was higher than in the adult cortex and was comparable to the cortex concentration on PND4.

### 3.5. TH Concentrations in the Brain Do Not Differ between Male and Female Pups

**Figure 3:**
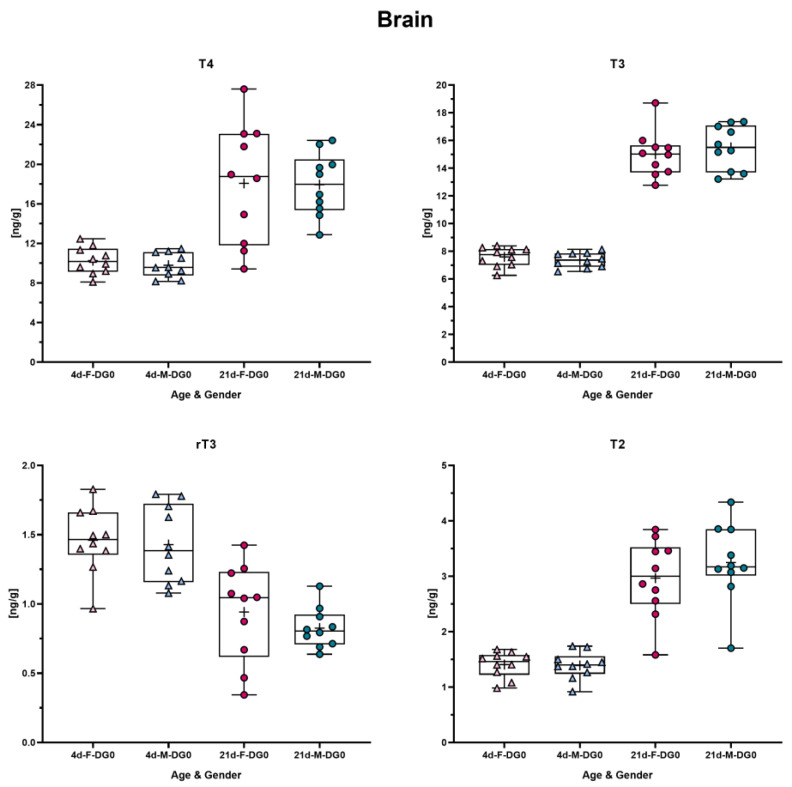
Comparison of TH concentration between male and female rats on PND4 and -21. Each graph shows concentration in male and female rat brain for each analyte and in each box plot the mean is marked with a “+”. In the presented data, the triangles depict PND4 and dots PND21; blueish color reflects male and reddish color female rats. The data were analyzed by unpaired Welch *t*-test to determine difference between male and female rats in each TH analyte concentration on PND4 and PND21, respectively. No statistically significant difference was observed.

The data from the same animals shown in the Section 3.3 were examined to determine whether male and female pups yield different concentrations in the brain. To prove this, an unpaired Welch *t*-test was conducted, and * *p* was set to ≤0.05. For none of the analytes was a statistical difference between male and female pups detected (Figure 3).

## 4. Discussion

The robust determination of TH alterations in an offspring brain can become a key parameter in assessing thyroid hormone disruption. This requires in particular a robust and validated analytical method as well as a historical control database of normal, undisturbed TH levels in the desired matrix. Based on such a historical control database, it is possible to reliably measure and assess potential adverse effects on TH levels after substance exposure. Here, we report on a validated xLC-MS/MS method for TH analysis in the brain, yielding LOQs of 0.02 ng/mL for T1, T2 and rT3 and 0.15 ng/mL for T3 and T4. Because of the utilization of a mixed sample preparation, in which we first performed protein precipitation followed by an additional clean-up of the samples via on-line HLB SPE cartridges, it was possible to develop a TH method for the brain matrix with high accuracy and precision. This method was validated by attaining accuracies at LOQ level between 90 and 110% with a corresponding precision of 1.8–9.1%. Most reports on TH levels in the brain focus on the analysis of T4 and T3 concentrations. Our method includes additional downstream metabolites, rT3, 3,3′-T2 and 3-T1; of these, rT3 and 3,3′-T2 concentrations were measured in all samples. No difference in TH concentration in the brain was observed between male and female rats at any examined age, which is in line with the available literature [9,17,18]. From PND4 to PND21, an increase in T4, T3 and T2 concentrations and a decrease in rT3 concentration were detected in the brain. TH availability in tissues is closely regulated by deiodinases 2 and 3 during development, as mentioned briefly in the Introduction. It has been shown that in fetal and prenatal rats, DIO3 (inactivating T4 to rT3) expression is high and declines postnatally, whereas DIO2 (activating T4 to T3) expression increases postnatally [19,20]. Therefore, we assume that this might be an explanation of decreased rT3 concentrations and increased T4, T3 and T2 concentrations on PND21 in rat brains.

In this study, TH concentrations were further separately analyzed in the cerebellum and cortex. In particular, TH alterations in the cerebellum during fetal and postnatal development are reported to cause intellectual impairments in humans [21]. In rodents, TH alterations during the first two postnatal weeks (in our study, TH was measured on PND4 and -21/22) could lead to impaired cerebellar function causing impaired motor coordination and balance. In this time, cerebellar morphogenesis such as Purkinje cell dendritogenesis and granule cell migration is affected by THs [22]. Investigation of THs in specific brain regions revealed that T4 concentration is similar within age groups in the cerebellum and cortex. T3 and T2 concentrations were lower in the cerebellum on PND4 compared to their values in the cortex, and on PND22, the T2 cerebellum concentration rose to a similar value as in the cortex. The rT3 concentrations followed an opposite pattern, i.e., a similar concentration in both regions on PND4 but a lower concentration in the cerebellum than in the cortex on PND22. When looking at the values in adult rats, there is a high standard deviation in T4 concentration in the cerebellum so that with these data, it cannot clearly be explained whether the T4 cerebellum concentration was higher or similar as in the cortex. The adult rT3 cerebellum concentration as analogous to its concentration on PND22, and the adult T3 concentration in each region was comparable to the value yielded in the cortex on PND4. Similar to the concentration in the cortex on PND4 was the T2 concentration found in the cortex of adult rats, whereas the T2 concentration in the adult cerebellum was slightly higher, as in adult cortex.

A comparison of the data here reported to the literature is difficult because of several factors: analysis of different brain regions, efficacy of extraction method, choice of analysis method, examination of different ages, and choice of rat species. One mentioned parameter is the region-specific brain analysis, for which the analysis of either the forebrain or the cortex is mostly found [10,14,23]. Whole-brain analysis is mainly performed when examining fetal pooled brain samples due to the limitation of the matrix [23]. Different extraction methods are another critical variable because they inter alia have different limitations and handling errors. The liquid–liquid extraction (LLE) method is time-consuming, and large amounts of solvent are needed. The method here presented has the advantage that the SPE unit is automated, and therefore, handling errors are negligible. Mixed sample preparation (protein precipitation and online SPE or LLE and SPE) might be prone to limitations too, since analyte loss could occur over the many procedure steps. However, the stable isotope-labeled internal standard was added early in the sample preparation to intercept analyte loss. Lastly, the analytical method chosen for the readout is influenced by its sensitivity and specificity in detecting the given analytes in a matrix, whereas enzyme-based assays might be more prone to impurities or influenced by the mode of action (MOA) of a substance than LC-MS/MS.

A summary of the reported TH concentrations in different brain regions in some of the literature is recorded in Table 3. No data comparable to the method here presented regarding analyzed matrix tissue or age have yet been published.

Additionally, the following table (Table 4) gives an overview of the method performance in the quantification of TH concentrations (LOQs and minimum quantification limits (MQLs)) in the brain reported in the literature. In accordance with the mentioned LOQs in Table 4, the defined LOQs for the brain used in this method were 0.02 ng/mL for T1, T2 and rT3 and 0.15 ng/mL for T3 and T4. In this publication, the reported accuracies ranged from 90 to 110% with a precision range of 1.8–9.1%. This is comparable to the published recoveries of 94.9–102% in brain tissue [9].

Besides the difference in region-specific brain analysis found in the literature, sample preparation reflects a big indicator of technical variation. The majority of the published methods for sample preparation are originally based on liquid–liquid extraction (LLE) using chloroform and MeOH followed by further purification with Bio-Rad AG 1 × 2 resin columns, as first reported by Morreale de Escobar et al. in 1985, whose method was based on previous descriptions by Folch et al. and Gordon et al. [26,27,28]. Over the years, sample preparation was further optimized by adjusting LLE parameters, using SPE cartridges instead of a resin column and optimizing the washing step during the SPE procedure to further remove phospholipids [9,11,23]. During method development in the study reported here, the described LLE was tested, compared to previously used sample preparations and evaluated regarding measured TH concentration and recovery of a spiked amount. No significant improvement using LLE was reached; therefore, sample preparation was finalized using the procedure described in Section 2.2.1. Brain pool replicates were included in each analysis to determine the technical variability of the used sample preparation. The derived RSDs were all below 20%, reflecting the low influence from technical variability of the sample preparation on the final calculated concentration. Therefore, it is assumed that the major influence on sample group variation is thus caused by biological differences between the animals. For a better estimation of technical variability in the future, technical replicates of matrix pool samples should be constantly generated and included in each analysis.

## 5. Conclusions

Taking all the mentioned aspects into account, it is concluded that the developed brain method guarantees a robust, reproducible and precise analysis of TH analytes in the brain matrix since it fulfills the required acceptance criteria [16]. The estimated technical variability of brain pool replicates was below 20%, underlining the previously mentioned statement about robustness. Thus, the presented method depicts a suitable diagnostic tool to detect decisive effect parameters for the investigation of thyroid hormone-related neurodevelopmental toxicity. To additionally determine what is a dose-related effect and what is biological variation, a brain-method-specific TH database will be generated by continuously analyzing control animals.

## Figures and Tables

**Table 1:** General MS parameters adjusted for the method.

MS/MS	Curtain Gas (CUR)	Collision Gas (CAD)	Ion Spray Voltage (IS)	Temperature (TEM)	Ion Source Gas 1 (GS1)	Ion Source Gas 2 (GS2)
Parameters	50	8	5500	600	50	55

**Table 2:** The table below lists the RSD in percentage calculated for each analyte in the brain matrix. The brain pool includes 12 technical replicates, although T2 was detected in only 9 out of 12.

RSD (%)/Analyte	T4	T3	rT3	T2
Brain pool (adult)	7.5	14.9	20	19.1

**Table 3:** Overview of concentration cited in the published literature. Analyzed brain region, determined concentration of THs (mainly T4 and T3) and age of rat are listed from each of the literature references.

Age of Rat	Brain Region	TH Concentration	Reference
PND12 males	Half brain	4 ng/g tissue T3	[11]
PND16 males	Hippocampus and cortex	7 ng/g and 6.2 ng/g tissue T3	[24]
Fetal GD20	Whole brain	0.6 ng/g T4 and 0.7 ng/g T3	[23]
PND14	Cortex	3.5 ng/g T4 and 7.9 ng/g T3	[23]
PND0 and 2	Forebrain	~0.8 ng/g T4 ~1 ng/g T3	[9]
PND6 and 14	One hemisphere	~1.5 ng/g and~3 ng/g T4 ~2 ng/g and~4 ng/g T3	[9]
PND2	Telencephalon	~0.5 ng/g T4 ~1.6 ng/g T3 ~0.075 ng/g rT3	[18]
Adult males	Cerebellum	~1500 fmol/g T3 ~3500 fmol/g T4	[7]

**Table 4:** Literature review of MQL and LOQ for TH measurement in rat brains. Besides MQL and LOQ, the used analytical method and corresponding analyte are listed.

Method	LOQ/MQL	Analytes	Reference
Stable isotope dilution LC-MS/MS	0.005 ng/g	T4 and T3	[23,25]
RIA	1.09 ng/mL 6.67 ng/dL	T4 T3	[24]
LC-MS/MS	0.105 ng/g 0.04 ng/g 0.09 ng/g	T3 rT3 T4	[9]
LC-MS/MS	0.01 ng/g 0.05 ng/g	T4 and rT3 T3	[18]

## Data Availability

The original contributions presented in this study are included in the article; further inquiries can be directed to the corresponding author/s. The data are not publicly available due to privacy restrictions.

## Supplementary Materials

The following supporting information can be downloaded at: https://www.mdpi.com/article/10.3390/metabo14010061/s1, Figure S1: HPLC chromatogram of analytes and their corresponding isotope labelled internal standard; Table S1: Overview of MRM transitions and MS parameters; Table S2: Listed information of determined method validation parameters.
