# Supplementary material for: Thyroid Hormone Metabolites Quantified in Pup and Adult Rat Cerebellum, Cortex and Whole-Brain Samples Using an Automated Online SPE-LC-MS/MS Method
Source: Metabolites. 2024 Jan 17;14(1):61. doi: 10.3390/metabo14010061 (PMC10820277; doi:10.3390/metabo14010061)
Supplement: Supplementary file 1 [file metabolites-14-00061-s001.zip › metabolites-2809592-supplementary.pdf]

metabolites

MDPI

Table S1. Overview of MRM transitions and MS parameters.

RSD [%]/Analyte	T4	T3	rT3	3,3′-T2	3-T1
Precursor Mass (m/z)	777.7	651.9	651.900	525.5	400
Quantifier Ion	731.6	478.7	605.701	479.9	354
Collision Energy (CE)	35	49	33	29	25

Figure S1. HPLC chromatogram of analytes and their corresponding isotope labelled internal standard.

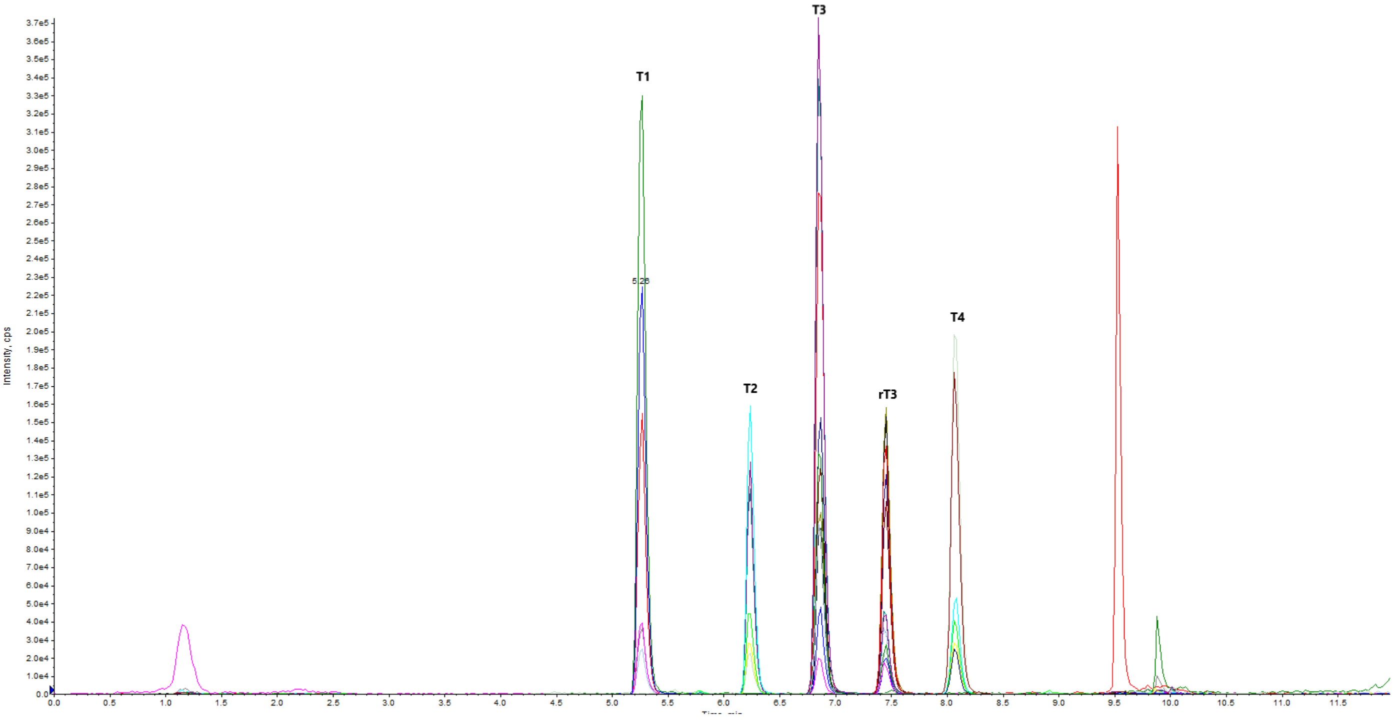

Table S2. Listed information of determined method validation parameters.

	T1	T2	rT3	T3	T4
**Quantifier**	256	480	606	479	732
**LOQ [ng/mL]**	0.02	0.02	0.02	0.15	0.15
**Accuracy [%]**	106	110	90	99	99
**Precision RSD [%]**	9.1	5.7	5	2.4	1.8
**10 x LOQ Accuracy [%]**	99	116	98	92	104
**10 x LOQ Precision RSD [%]**	3.7	3.9	3.6	3	4.2
**Calibration range [ng/mL]**	0.01-0.3	0.005-0.5	0.005-0.3	0.05-2	0.05-4
**Specificity**	N/A	N/A	N/A	N/A	4.9
